# Control of βAR- and *N*-methyl-*D*-aspartate (NMDA) Receptor-Dependent cAMP Dynamics in Hippocampal Neurons

**DOI:** 10.1371/journal.pcbi.1004735

**Published:** 2016-02-22

**Authors:** Andrew Chay, Ilaria Zamparo, Andreas Koschinski, Manuela Zaccolo, Kim T. Blackwell

**Affiliations:** 1 Molecular Neuroscience Department, Krasnow Institute, George Mason University, Fairfax, Virginia, United States of America; 2 Venetian Institute of Molecular Medicine, Padova, Italy; 3 Department of Physiology, Anatomy and Genetics, Oxford University, Oxford, United Kingdom; École Normale Supérieure, College de France, CNRS, FRANCE

## Abstract

Norepinephrine, a neuromodulator that activates β-adrenergic receptors (βARs), facilitates learning and memory as well as the induction of synaptic plasticity in the hippocampus. Several forms of long-term potentiation (LTP) at the Schaffer collateral CA1 synapse require stimulation of both βARs and *N*-methyl-*D*-aspartate receptors (NMDARs). To understand the mechanisms mediating the interactions between βAR and NMDAR signaling pathways, we combined FRET imaging of cAMP in hippocampal neuron cultures with spatial mechanistic modeling of signaling pathways in the CA1 pyramidal neuron. Previous work implied that cAMP is synergistically produced in the presence of the βAR agonist isoproterenol and intracellular calcium. In contrast, we show that when application of isoproterenol precedes application of NMDA by several minutes, as is typical of βAR-facilitated LTP experiments, the average amplitude of the cAMP response to NMDA is attenuated compared with the response to NMDA alone. Models simulations suggest that, although the negative feedback loop formed by cAMP, cAMP-dependent protein kinase (PKA), and type 4 phosphodiesterase may be involved in attenuating the cAMP response to NMDA, it is insufficient to explain the range of experimental observations. Instead, attenuation of the cAMP response requires mechanisms upstream of adenylyl cyclase. Our model demonstrates that Gs-to-Gi switching due to PKA phosphorylation of βARs as well as Gi inhibition of type 1 adenylyl cyclase may underlie the experimental observations. This suggests that signaling by β-adrenergic receptors depends on temporal pattern of stimulation, and that switching may represent a novel mechanism for recruiting kinases involved in synaptic plasticity and memory.

## Author Summary

Noradrenaline is a stress related molecule that facilitates learning and memory when released in the hippocampus. The facilitation of memory is related to modulation of synaptic plasticity, but the mechanisms underlying this modulation are not well understood. We utilize a combination of live cell imaging and computational modeling to discover how noradrenergic receptor stimulation interacts with other molecules, such as calcium, required for synaptic plasticity and memory storage. Though prior work has shown that noradrenergic receptors and calcium interact synergistically to elevate intracellular second messengers when combined simultaneously, our results demonstrate that prior stimulation of noradrenergic receptors inhibits the elevation of intracellular second messengers. Our results further demonstrate that the inhibition may be caused by the noradrenergic receptor switching signaling pathways, thereby recruiting a different set of memory kinases. This switching represents a novel mechanism for recruiting molecules involved in synaptic plasticity and memory.

## Introduction

Long-term potentiation (LTP) in the hippocampus has long been studied as a mechanism underlying mammalian learning and memory. At least two mechanistically distinct phases of LTP have been characterized, including an early-phase LTP, which decreases over the course of two hours, and a late-phase (L-LTP), which endures for more than two hours. Though 1 s of 100 Hz electrical stimulation of Schaffer collaterals produces only early-phase LTP in area CA1, pretreatment of β-adrenergic receptors (βARs) with the selective agonist isoproterenol followed by 1s of 100 Hz electrical stimulation produces L-LTP [1–5]. Similarly, 3 min of 5 Hz stimulation fails to induce LTP by itself, but in the presence of isoproterenol, the same stimulation induces robust L-LTP [2–6], often called β-LTP. Because L-LTP resembles memory storage in its requirement for protein synthesis, understanding the role of βARs in producing L-LTP may shed light on molecular mechanisms of memory storage.

**Fig 1:**
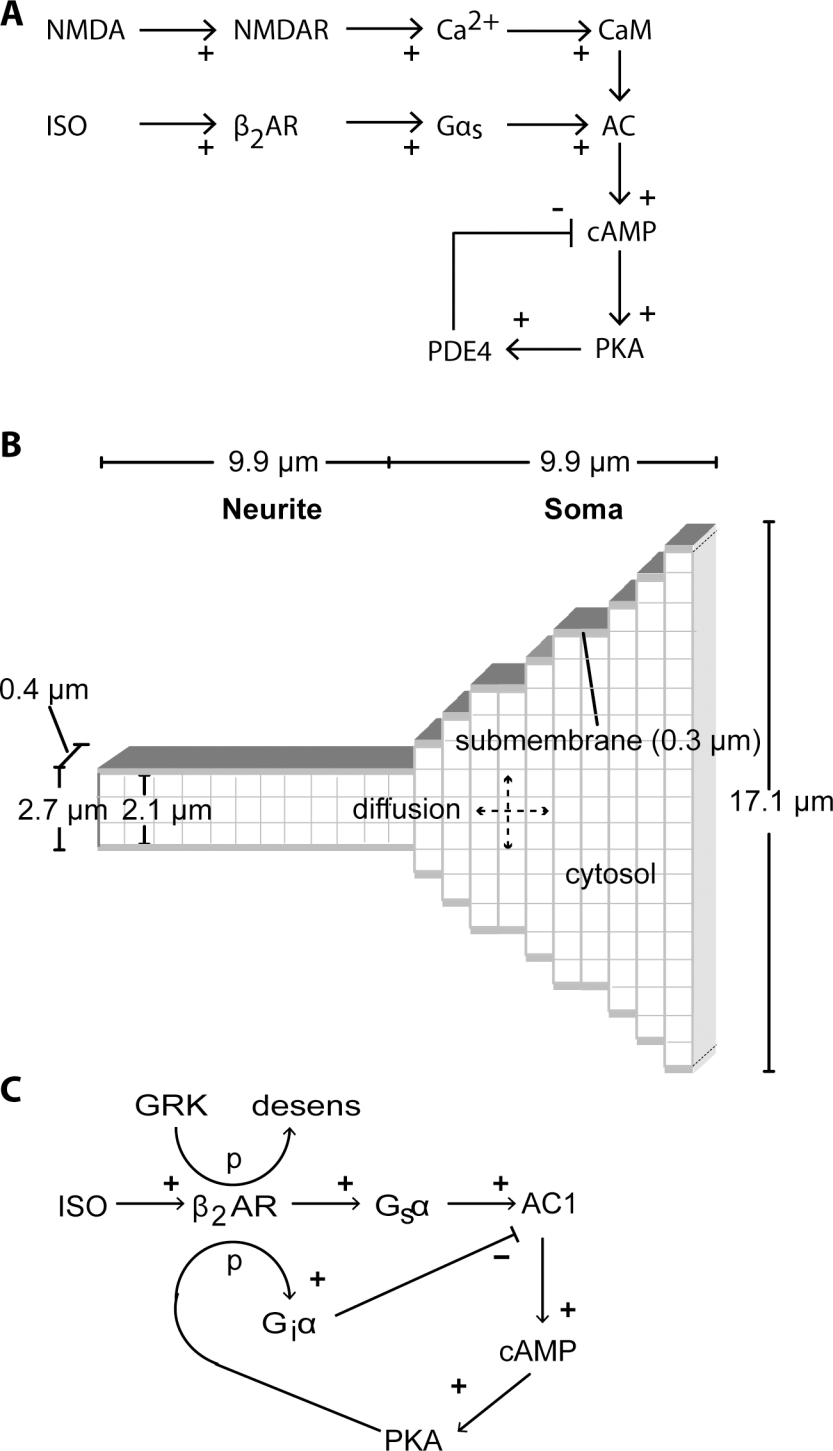
Computational model of βAR- and NMDAR-mediated cAMP signaling pathways. **A.** Signaling pathways leading to cAMP production, and the downstream mechanisms involving PDE4. **B.** Morphology implemented for the computational model. Reflective boundary conditions occur at the membrane as well as cut surface of the soma and dendrite. The morphology is discretized with 0.9 μm voxels for the cytosol, with one layer of 0.3 μm submembrane voxels and one layer of 0.6 μm voxels adjacent to the submembrane voxels. **C.** Signaling pathways involved in Gs-Gi switching and GRK desensitization implemented in the model. *p* indicates a phosphorylation step.

Several studies have identified molecular components involved in β-LTP [2–5]. Both βARs and the requisite calcium influx through *N*-methyl-*D*-aspartate receptors (NMDARs) couple to the cAMP signaling pathway, though via different intermediaries (Fig 1A). The calcium (bound to calmodulin) stimulates adenylyl cyclase types 1 and 8 (AC1 and AC8) which catalyze cAMP production [7,8]. Stimulation of βARs activates the Gs subtype of GTP binding protein, which stimulates adenylyl cyclase isoforms [9]. AC1 and AC8, abundantly expressed in CA1 pyramidal neurons [10–13], are required for L-LTP induction and long-term memory [14]. The synergistic activation of AC1 by simultaneous Ca^2+^ and Gs signals in AC1-expressing HEK293 cells [15] as well as synergistic cAMP-mediated transcription in cultured hippocampal neurons [16] suggests that NMDA and isoproterenol would enhance cAMP production during β-LTP, but this has not been demonstrated.

The regulation of cAMP downstream of adenylyl cyclases is largely carried out by phosphodiesterases (PDEs), which are regulated by cAMP-dependent kinase (PKA). Type 4 PDEs (PDE4) comprise the major cAMP-degrading PDE family in the hippocampus [17]. PKA phosphorylation of PDE4s increases their activity [18,19] forming a cAMP-PKA-PDE4 negative feedback loop, which is a significant contributor to cAMP signaling dynamics downstream of βARs [20,21].

To investigate how NMDARs and βARs contribute to the cAMP and PKA underlying β-LTP, we combined FRET-based live-cell imaging of cAMP in cultured rat hippocampal neurons with a spatial mechanistic model of the cAMP signaling network in a hippocampal CA1 pyramidal neuron. Unexpectedly, when NMDA was applied after the onset of isoproterenol in experiments, rather than generate synergistic elevations of cAMP, the cAMP was attenuated compared to that of NMDA alone. This attenuation of NMDA-induced cAMP following isoproterenol was not sufficiently explained by either PKA or PDE4 in the model. Instead, our results suggest that PKA-mediated Gs-Gi switching following βAR activation may underlie the attenuation of NMDA-induced cAMP following isoproterenol pretreatment.

## Materials and Methods

### Experiments

Primary hippocampal cell cultures were prepared from brains of E18 Sprague Dawley rats as previously described [22]. Briefly, surgically dissected hippocampi were enzymatically and mechanically dissociated and the resultant cell suspensions were plated on coverslips coated with poly-L-lysine (Sigma) and maintained in Neurobasal medium (Invitrogen) supplemented with B27 (Invitrogen). The medium was partially changed once a week. At 5–9 days in vitro (the day before the experiments) neurons were transiently transfected with the Epac1 based FRET sensor for cAMP [23] using Transfectin (Biorad) transfection reagent.

The experiments were performed on an inverted Olympus IX 70 microscope using a 60xNA, 1.4 oil-immersion objective. The microscope was equipped with a CCD camera (Sensicam QI, PCO, U.S.A.), a software-controlled monochromator (Polychrome IV, TILL Photonics, Germany), and an optical beam-splitter device (Multispec Microimager; Optical Insights, U.S.A.). All filters and dichroics were from Chroma Technology. Live images were acquired for 200–300 ms at 3 s intervals.

The day of the experiment, coverslips were mounted in an imaging chamber at room temperature and maintained in a modified Hank’s balanced salt solution (HBSS) as follows: 137 mM sodium gluconate, 5 mM potassium gluconate, 0.6 mM Na_2_HPO_4_, 0.6 mM KH_2_PO_4_, 5.5 mM glucose, 20 mM HEPES, 1.4 mM calcium gluconate pH 7.4 (gluconate was used to replace chloride to avoid the unequal quenching of CFP and YFP due to chloride ion entry during NMDA stimulation). Images were acquired using TILLvisION v3.3 software and then processed off-line using ImageJ. Cells received either the NMDA alone stimulation, or the NMDA after ISO stimulation, both for control experiments, and in the presence of either H89 or rolipram. When isoproterenol was pre-applied, the NMDA was then applied between 2 and 5 minutes later, after the response to isoproterenol reached a plateau. FRET changes were measured as changes in the background-subtracted 480/545 nm fluorescence emission intensities on excitation at 430 nm and expressed as *R*/*R*_0_, where *R* is the ratio at time *t* and *R*_0_ is the ratio at time = 0 s. The amplitude of response was calculated as *ΔR/R*_*0*_, where *ΔR = R–R*_*0*_ and expressed in bar graphs as % FRET ratio change (%ΔR/R_0_). All data are presented as means and SEM. Student’s t tests (two-tailed) were performed using SAS (SAS Institute) to evaluate statistical significance (*P* ≤ 0.05). When variances were unequal, the Satterthwaite method for variances of the samples was used.

Pharmacological stimuli, *N*-methyl-*D*-aspartic acid (NMDA, 300 μM), Glycine (3 μM), 3-Isobutyl-1-methylxanthine (IBMX, 100 μM), isoproterenol (ISO, 1 μM), rolipram (1 μM), dopamine (20 μM), H-89 dihydrochloride hydrate (H89, 10 μM), all from Sigma, were prepared in stocks and diluted to the final concentration (indicated in brackets) in the bath.

### Spatial mechanistic model

**Fig 2:**
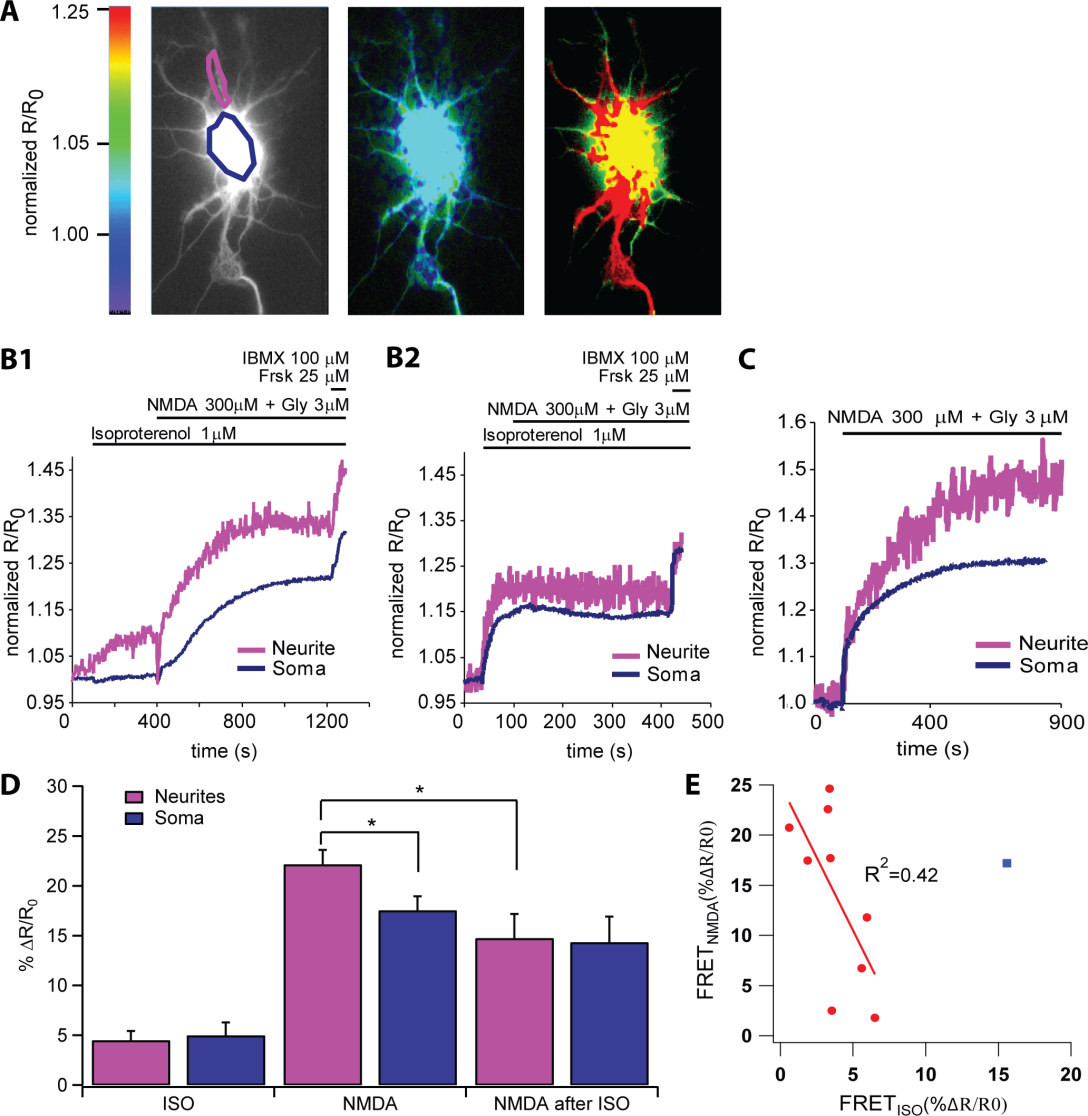
Experimental responses to isoproterenol and/or NMDA in cultured hippocampal neurons. **A.** Sample images of a cultured hippocampal neuron showing sensor localization (left) and ratio images before and during stimulation with NMDA (center and right). The saturating concentration of NMDA (300 μM) was based on [59,60]. The image on the left is used to mark the regions of interest for calculating the FRET responses in neurites (magenta) and soma (blue). The middle and right pseudocolored FRET ratio images show the FRET ratios before (t = 0s) and after (t = 350s) forskolin application, respectively. The color scale bar indicates the intensity of the calculated FRET response (normalized R/R_0_). **B.** Sample traces of the cAMP response to the NMDA after isoproterenol stimulus showing the variability of the response. **B1:** trace demonstrating a large cAMP response to NMDA following a relatively small cAMP response to isoproterenol. **B2:** trace demonstrating a complete block of NMDA-induced cAMP following a relatively large cAMP response to isoproterenol. **C.** Sample trace of the cAMP response to NMDA alone. **D.** Averaged cAMP responses to isoproterenol alone (ISO; *n* = 10), NMDA alone (*n* = 46), and the NMDA after ISO stimulus (*n* = 10). Note that the ISO alone response is taken from the ISO part of the NMDA after ISO traces, and the NMDA after ISO response is the NMDA-induced portion of the cAMP response, with the ISO-induced response subtracted from the peak response. Data represent the means and SEM. * denotes 0.01 < *p* < 0.05. In the soma, the *total* NMDA after ISO response of (%ΔR/R_0_ = 19.3, not shown in figure) is slightly less than the sum of the NMDA (%ΔR/R_0_ = 17.5) + ISO (%ΔR/R_0_ = 5.0) responses. In the neurite, the *total* NMDA after ISO (%ΔR/R_0_ = 19.2, not shown in figure) is much less than the sum of the NMDA (%ΔR/R_0_ = 22.2) and ISO (%ΔR/R_0_ = 4.5) responses. **E.** ECorrelation of the somatic cAMP responses to NMDA vs. the cAMP responses to isoproterenol. When a single outlier is excluded, the negative correlation is strong (R^2^ = 0.4209). Note that forskolin (Frsk, 25 μM) and IBMX (100 μM) were added at the end of each experiment to attain maximal FRET signals.

We created a spatial, mechanistic model of the NMDAR and βAR activated signaling pathways in hippocampal CA1 pyramidal neurons by modifying an existing model of the signaling pathways underlying L-LTP (Tables 1–3). The morphology of the model represents the region of interest of the cultured hippocampal neurons used for imaging (Fig 2A). Thus, we modeled one neurite and half of the soma (for computational efficiency, Fig 1B) with diameters based on the morphology of the cultured neurons. These values are similar to that reported for the apical dendrite and soma of reconstructed neurons in NeuroMorpho.org. The morphology is discretized with 0.9 μm voxels for the cytosol, with one layer of 0.3 μm submembrane voxels and one layer of 0.6 μm voxels adjacent to the submembrane voxels. A subset of the molecules in the system is diffusible (Table 3). Membrane associated proteins, such as βAR, G proteins, AC1, AC8 and PKA holoenzyme are organized as multi-protein complexes by A-Kinase-Anchoring-Proteins (AKAPs) [24,25]. The AKAPs are made implicit in this model by colocalizing at the membrane the molecules comprising these multi-protein complexes. In addition, for most simulations the PDE4s were considered anchored [26], and thus do not diffuse.

**Table 1:** Reactions and rate constants of signaling pathways in the model.

Reaction	kf (nM^-1^ ms^-1^)	kb (ms^-1^)	kcat (ms^-1^)	Source
Isoproterenol + R ↔ Iso-R	5.556E-06	0.005		[27,28]
Iso-R + Gsαβγ ↔ Iso-R-Gsαβγ → Iso-R-Gβγ + GsαGTP	6.000E-07	1.00E-06	0.02	[29]
R + Gsαβγ ↔ R-Gsαβγ	4.000E-08	3.00E-07		PMR
Isoproterenol + R-Gsαβγ ↔ Iso-R-Gsαβγ → Iso-R-Gβγ + GsαGTP	2.500E-06	5.00E-04	0.02	[27,28]
Iso-R-Gβγ → Iso-R + Gβγ	0.08			#
GsαGTP → GsαGDP	0.001			[30,31]
GsαGDP + Gβγ → Gsαβγ	0.1			[32]
PMCA + Ca ↔ PMCA-Ca → PMCA + CaOut	5.000E-05	0.007	0.0035	[33]
NCX + Ca ↔ NCX-Ca → NCX + CaOut	1.680E-05	0.0112	0.0056	[34,35]
Leak + Ca_Ext_ ↔ Leak-Ca_Ext_ → Leak + Ca	1.500E-06	0.0011	0.0011	adj
Calbindin + Ca ↔ Calbindin-Ca	2.800E-05	0.0196		[36]
2 Ca + CaM ↔ CaMCa_2_	6.000E-06	0.0091		[37]
2 Ca + CaMCa_2_ ↔ CaMCa_4_	0.0001	1		[38]
AC1 + GsαGTP ↔ AC1-GsαGTP	3.850E-05	0.01		[39]
AC1 + CaMCa_4_ ↔ AC1-CaMCa_4_	6.000E-06	0.0009		[40]
AC1-GsαGTP + CaMCa_4_ ↔ AC1-GsαGTP-CaMCa_4_	6.000E-06	0.0009		[41]
AC1-GsαGTP-CaMCa_4_ + ATP ↔ AC1-GsαGTP-CaMCa_4_-ATP → AC1-GsαGTP-CaMCa_4_ + cAMP	1.000E-05	2.273	0.05684	[16,41]
AC1-CaMCa_4_ + ATP ↔ AC1-CaMCa_4_-ATP → AC1-CaMCa_4_ + ATP	1.000E-05	2.273	0.005684	[41]
AC8 + CaMCa_4_ ↔ AC8-CaMCa_4_	1.250E-06	0.001		[40]
AC8-CaMCa_4_ + ATP ↔ AC8-CaMCa_4_-ATP → AC8-CaMCa_4_ + ATP	1.000E-05	2.273	0.00284	[42]
PDE1 + CaMCa_4_ ↔ PDE1-CaMCa_4_	0.0001	0.001		[43]
PDE1-CaMCa_4_ + cAMP ↔ PDE1-CaMCa_4_-cAMP → PDE1-CaMCa_4_ + AMP	4.600E-06	0.044	0.011	[44]
PP2B + CaM ↔ PP2B-CaM	4.600E-06	1.200E-06		PMR
PP2B + CaMCa_2_ ↔ PP2B-CaMCa_2_	4.600E-05	1.200E-06		PMR
PP2B + CaMCa_4_ ↔ PP2B-CaMCa_4_	4.600E-05	1.200E-06		[45]
PP2B-CaM + 2 Ca ↔ PP2B-CaMCa_2_	6.000E-06	9.100E-04		[46]
PP2B-CaMCa_2_ + 2 Ca ↔ PP2B-CaMCa_4_	0.0001	1		[47]
CaMKII + CaMCa_4_ ↔ CaMKII-CaMCa_4_	1.000E-05	0.003		[48]
CaMKII-CaMCa_4_ + CaMKII-CaMCa_4_ ↔ Complex	1.000E-07	0.01		&
p-CaMKII-CaMCa_4_ + CaMKII-CaMCa_4_ ↔ p-Complex	1.000E-07	0.01		&
p-CaMKII-CaMCa_4_ + Complex → p-CaMKII-CaMCa_4_ + p-Complex	1.000E-07			&
CaMKII-CaMCa_4_ + Complex → CaMKII-CaMCa_4_ + p-Complex	1.000E-07			&
Complex + Complex ↔ Complex + p-Complex	1.000E-05			&
Complex + p-Complex ↔ p-Complex + p-Complex	3.000E-05			&
p-CaMKII-CaMCa_4_ ↔ CaMCa_4_ + p-CaMKII	8.000E-07	1.000E-05		[48]
p-CaMKII + PP1 ↔ p-CaMKII-PP1 → CaMKII + PP1	6E-10	3.400E-04	0.000086	[49]
p-CaMKII-CaMCa_4_ + PP1 ↔ p-CaMKII-CaMCa_4_-PP1 → CaMKII-CaMCa_4_ + PP1	6E-10	3.400E-04	0.000086	[49]
PKA + 2 cAMP ↔ PKAcAMP_2_	8.695E-08	2.000E-05		[50,51]
PKAcAMP_2_ + 2 cAMP ↔ PKAcAMP_4_	1.154E-07	0.0002		[50,51]
PKAcAMP_4_ ↔ PKAr + 2 PKAc	1.600E-06	1.700E-07		[52]
Epac1camps + cAMP ↔ Epac1camps-cAMP	3.300E-08	8.000E-05		[23]
I1 + PKAc ↔ I1-PKAc → Ip35 + PKAc	1.400E-06	0.0056	0.0014	[53]
Ip35 + PP1 ↔ Ip35-PP1	1.000E-06	1.100E-06		[54,55]
Ip35 + PP2B ↔ Ip35-PP2B → I1 + PP2B	2.330E-06	0.0112	0.0028	[56,57]
Ip35-PP1 + PP2B ↔ Ip35-PP1-PP2B → I1 + PP1-PP2B	2.330E-06	0.0112	0.0028	[56,57]
PP1-PP2B → PP1 + PP2B	0.0015			#
AMP → ATP	0.001			
PDE4 + cAMP ↔ PDE4-cAMP → PDE4 + AMP	2.166E-05	0.06895	0.017233	[58]
PDE4 + PKAc ↔ PDE4-PKAc → pPDE4 + PKAc	4.280E-07	0.00056	0.000125	[19]
pPDE4 + cAMP ↔ pPDE4-cAMP → pPDE4 + AMP	4.332E-05	0.1379	0.034467	[19]
PDE4-cAMP + PKAc ↔ PDE4-cAMP-PKAc → pPDE4-cAMP + PKAc	4.280E-07	0.00056	0.000125	[19]
pPDE4 → PDE4	2.500E-06			adj

PMR: principal of microscopic reversibility. Adj: adjusted to yield desired basal concentration. Two types of reactions were added because NeuroRD is restricted to first or second order reactions: &:CaMKII phosphorylation reactions involving Complex are required to produce the observed calcium sensitivity, and capture the probability that two calmodulin bound CaMKII subunits are adjacent in the holoenzyme.

#: Rapid dissociation after enzyme reaction prevents accumulation of these intermediate forms.

**Table 2:** Initial concentrations of molecule species in the model. Molecules not listed have initial concentrations of 0. All membrane bound molecules have zero concentration in the cytosol, and are specified as membrane densities.

**Molecule**	**Dendrite (nM)**	**Soma (nM)**
Isoproterenol	4	4
Ca	49	49
Ca_Ext_	2014068	2014068
Calbindin	150148	150148
Ca-Calbindin	10790	10790
Epac1camps	1950	1950
Epac1camps-cAMP	50	50
ATP	1,998,638	1,998,638
cAMP	27	27
AMP	495	289
PDE1	3457	3457
PDE1-CaMCa_4_	489	489
PDE1-CaMCa_4_-cAMP	1	1
CaM	8775	8775
CaMCa_2_	291	291
CaMCa_4_	1	1
PP2B-CaM	2990	2990
PP2B-CaMCa_2_	989	989
PP2B-CaMCa_4_	4	4
CaMKII	18710	18710
CaMKII-CaMCa_4_	87	87
p-CaMKII-CaMCa_4_	1092	1092
p-CaMKII	106	106
p-CaMKII-PP1	3	3
p-CaMKII-CaMCa_4_-PP1	3	3
PKAc	14	14
I1	507	507
I1-PKAc	2	2
Ip35	5	5
PP1	556	556
Ip35-PP1	908	908
PDE4	2,766	1,749
PDE4cAMP	34	10
PKAcPDE4	25	16
pPDE4	122	81
pPDE4-cAMP	1	1
**Membrane Molecule**	**Dendrite sm (mol/μm**^**2**^**)**	**Soma sm (mol/μm**^**2**^**)**
R	9.6	9.6
Iso-R	0.6	0.6
R-Gsαβγ	354.6	354.6
Iso-R-Gsαβγ	0.6	0.6
Gsαβγ	1827	1827
GsαGTP	0.6	0.6
Gβγ	59.4	59.4
Leak	376.2	376.2
PMCA	30	30
PMCA-Ca	7.8	7.8
NCX	1353	1353
NCX-Ca	66	66
AC1	1668.6	1668.6
AC1-GsαGTP	26.4	26.4
AC1-GsαGTP-CaMCa_4_	0.6	0.6
AC1-GsαGTP-CaMCa_4_-ATP	4.8	4.8
AC1-CaMCa_4_	14.4	14.4
AC1-CaMCa_4_-ATP	142.2	142.2
AC8	1825.2	1825.2
AC8-CaMCa_4_	3	3
AC8-CaMCa_4_-ATP	28.2	28.2
PKA	1763.4	1763.4
PKA-cAMP_2_	342.6	342.6
PKA-cAMP_4_	10.2	10.2
PKAr	9.6	9.6

sm: submembrane

**Table 3:** Diffusion constants for diffusible molecules in the model.

Molecule	D_coeff_ (um^2^ s^-1^)
Isoproterenol	111.3
Ca	174.3
Ca_Ext_	174.3
Calbindin	9.3
Calbindin-Ca	9.3
Epac1camps	10
Epac1camps-cAMP	10
ATP	74.7
cAMP	86.4
AMP	85.5
CaM	11
CaMCa_2_	11
CaMCa_4_	11
CaMKII	3.6
CaMKII-CaMCa_4_	3.6
p-CaMKII-CaMCa_4_	3.6
p-CaMKII	3.6
PKAc	8.1
I1	10.6
I1-PKAc	10.6
Ip35	10.6

One set of simulations (dynamic recruitment of PDE4s) required additional biochemical reactions (Table 4). Dynamic recruitment of PDE4 to cell membranes occurs in an activity-dependent manner [61], but the specific mechanisms by which PDE4 is recruited to the membrane are unknown, though may involve β-arrestin. In the model, we assume that a fraction of the PDE4 (called PDE4D) is anchored to a non-diffusible cytosolic anchoring protein (AP_cyt_) but is released by a cooperative, cAMP-dependent mechanism. The released PDE4D diffuses and binds to an anchoring protein localized to the submembrane (AP_sm_), thus completing the recruitment. In the dynamic recruitment simulations, one third of the total PDE4 was the PDE4D form. To demonstrate that the results are not dependent on the cAMP-dependent release mechanism, we ran additional simulations using elevation of Gsβγ instead of cAMP as the trigger. Though the Gsβγ trigger prevented PDE4D recruitment in response to NMDA, neither of these dynamic recruitment mechanisms could account for the reduction in the NMDA response after ISO pretreatment.

**Table 4:** Reactions and rate constants for dynamic recruitment in the model. X indicates cAMP bound or not to the catalytic site, in addition, these reactions occur for both the phosphorylated and unphosphorylated PDE4D forms. Y indicates PDE4D bound or not to AP. Z indicates PDE4D bound or not to AP_cyt_. AP_cyt_ is the unidentified anchoring protein for PDE4D in the cytosol, and AP_sm_ is the submembrane anchoring protein. AP_sm__PDE4D is the plasma membrane PDE4D in Fig 5.

Reaction	kf (nM^-1^ ms^-1^)	kb (ms^-1^)	kcat (ms^-1^)	source
AP_cyt__PDE4D_X + 2 cAMP ↔ AP_cyt_cAMP_2__PDE4D_X	8.70E-08	0.00002		adj
AP_cyt_cAMP_2__PDE4D_X + 2 cAMP ↔ AP_cyt_cAMP_4__PDE4D_X	1.15E-07	0.0002		adj
AP_cyt_cAMP_4__PDE4D_X ↔ AP_cyt_cAMP_4_ + PDE4D_X	1.60E-05	1.70E-06		adj
PDE4D_X + AP_sm_ ↔ AP_sm__PDE4D _X	1.70E-06	1.60E-05		adj
PKAc + Y_PDE4D_X ↔ Y_PKAcPDE4D_X → Y_pPDE4D_X + PKAc	4.28E-07	0.00056	0.000125	same as other PDE4
Z_PDE4D + cAMP ↔ Z_PDE4DcAMP → Z_PDE4D + AMP	2.166E-05	0.06895	0.017233	same as other PDE4
Z_pPDE4D + cAMP ↔ Z_pPDE4DcAMP → Z_pPDE4D + AMP	4.332E-05	0.1379	0.034467	same as other pPDE4
AP_sm__PDE4D + cAMP ↔ AP_sm__PDE4DcAMP →AP_sm__PDE4D + AMP	2.166E-04	0.6895	0.17233	10x case in Fig 5

adj: adjusted to effect

For the final set of simulations, the biochemical reactions of the signaling pathways were modified by adding receptor desensitization and Gs-Gi switching (Fig 1C), [62,63]. Table 5 provides the rate constants governing PKA phosphorylation of βARs, followed by activation of the Gi subtype of GTP binding protein. A single phosphorylation event decouples the βAR from Gs, but only the fully phosphorylated βAR can bind Gi. We assume βARs are phosphorylated with cooperativity and in a distributive manner, i.e. with PKA dissociating from the receptor after each phosphorylation event, which together enable an ultrasensitive response [64–66]. For most simulations, βARs require PKA phosphorylation at 4 sites [67] for Gi binding; however, for a subset of simulations, only 2 sites were phosphorylated to produce switching. The rates of Gi activation and hydrolysis were adjusted to produce a low basal quantity of GiαGTP. The reactions and kinetics for binding of GiαGTP to AC1 were derived from [68].

**Table 5:** Reactions and rate constants for PKA-mediated desensitization and switching in the model.

Reaction	kf (nM^-1^ ms^-1^)	kb (ms^-1^)	kcat (ms^-1^)	Source
Iso-R + PKAc ↔ Iso-R-PKAc → p-Iso-R + PKAc	3.424E-06	0.00448	0.001	Adj
p-Iso-R + PKAc ↔ p-Iso-R-PKAc → pp-Iso-R + PKAc	3.424E-06	0.00448	0.001	Adj
pp-Iso-R + PKAc ↔ pp-Iso-R-PKAc → ppp-Iso-R + PKAc	3.424E-05	0.00448	0.001	Adj
ppp-Iso-R + PKAc ↔ ppp-Iso-R-PKAc → pppp-Iso-R + PKAc	0.003424	0.00448	0.001	Adj
pppp-Iso-R + Giαβγ ↔ pppp-Iso-R-Giαβγ → pppp-Iso-R-Gβγ + GiαGTP	0.0006	0.001	0.00025	Adj
pppp-Iso-R-Gβγ → pppp-Iso-R + Gβγ	0.00025			Adj
pppp-Iso-R → ppp-Iso-R	2.500E-06			[69]
ppp-Iso-R → pp-Iso-R	2.500E-06			[69]
pp-Iso-R → p-Iso-R	2.500E-06			[69]
p-Iso-R → Iso-R	2.500E-06			[69]
GiαGTP → GiαGDP	0.000125			[31]
GiαGDP + Gβγ → Giαβγ	0.00125			[32]
AC1-CaMCa_4_ + GiαGTP ↔ AC1-GiαGTP-CaMCa_4_	6.250E-05	0.01		[68,70]
AC1-GiαGTP-CaMCa_4_ + ATP ↔ AC1-GiαGTP-CaMCa_4_-ATP → AC1-GiαGTP-CaMCa_4_ + cAMP	1.00E-05	2.273	0.0005684	[68]
AC1-GsαGTP + GiαGTP ↔ AC1-GsαGTP-GiαGTP	6.250E-05	0.01		[68,70]
AC1-GsαGTP-GiαGTP + CaMCa_4_ ↔ AC1-GsαGTP-GiαGTP-CaMCa_4_	6.00E-06	9.00E-04		
AC1-GsαGTP-GiαGTP-CaMCa_4_ + ATP ↔ AC1-GsαGTP-GiαGTP-CaMCa_4_-ATP → AC1-GsαGTP-GiαGTP-CaMCa_4_ + cAMP	1.00E-05	2.273	0.005684	[68]

Adj: Adjusted to effect

### Model simulation

Bath application of NMDA alone was simulated by injecting calcium, at t = 50s at a rate of 5 molecules per ms for 500 s, to create an intracellular Ca^2+^ concentration of ~1.4 μM [60]. Bath application of isoproterenol was simulated by injecting isoproterenol at t = 50s, at a rate of 2.15 molecules per ms for 1 s, to create an isoproterenol concentration of 1.0 μM. For a subset of simulations, to create higher or lower concentrations of isoproterenol, a higher or lower injection rate was used. Bath application of NMDA after ISO applied the isoproterenol stimulus at 50s, followed by the NMDA calcium stimulus at 170s.

The signaling pathways are simulated using a well-validated, efficient, mesoscopic stochastic reaction-diffusion algorithm, NeuroRD [71], version 2.1.9. Thus, the noise and fluctuations in the simulations are caused by the stochastic simulation technique. Because of this stochastic variability, some simulations are repeated to generate means and SEM. All simulations use a time step of 2.5 μs. Simulation output is processed using NRDPost (to calculate average concentration for defined regions in the morphology). The simulation and output processing software and the files used for the model simulations (S1 File) are freely available from modelDB (http://senselab.med.yale.edu/modeldb/showmodel.cshtml?model=184731).

### Data analysis

The responses in bar graphs for both model and experiments were calculated as follows. The response to NMDA alone was measured as the peak response to NMDA application alone. The response to ISO was measured as the peak response to ISO detected prior to NMDA application. The NMDA after ISO response was the peak response to NMDA, detected after both NMDA and ISO application, minus the ISO response during the 10 sec immediately prior to NMDA application. For all cases, the peak response was the mean value measured during a 10 sec window surrounding the peak. A synergistic effect implies that the peak response to NMDA after ISO is larger than the sum of the ISO response plus NMDA alone response. Equivalently, synergy is suggested if the mean NMDA after ISO response plotted in the graphs is larger than the response to NMDA alone. Peak responses for experiments are tabulated in S1 Data.

## Results

### Isoproterenol pretreatment can attenuate NMDA-induced cAMP

In CA1 pyramidal neurons, pretreatment of βARs facilitates several NMDAR-dependent forms of LTP [1–5,72]. The mechanism underlying the facilitation is suggested by previous research demonstrating that the catalytic activity of AC1 increases synergistically when GsαGTP and Ca^2+^/calmodulin signals coincide [15,16], but a synergistic increase in cAMP has not been demonstrated in hippocampal neurons. To investigate the effects of βAR and NMDAR interactions underlying βAR-dependent L-LTP, we performed live-cell imaging of cAMP in cultured hippocampal neurons expressing the FRET sensor Epac1-camps (Fig 2A). To approximate βAR activation followed by electrical stimulation, we bath applied isoproterenol and added NMDA when the isoproterenol-induced FRET change reached a plateau (henceforth called the NMDA after ISO stimulus).

FRET imaging of cAMP did not reveal a synergistic increase in cAMP in response to NMDAR and βAR stimulation. Isoproterenol by itself induced relatively weak cAMP responses that were similar in amplitude in the neurites and soma (*n* = 10, P = 0.648; Fig 2D). NMDA alone induced relatively robust cAMP responses, with average responses in neurites significantly higher than those in the soma (*n* = 46, P<0.0001; Fig 2C and 2D). However, when the NMDA was applied after the ISO stimulus, a synergistic response was not observed. In some neurons, isoproterenol pretreatment led to an NMDA-induced cAMP response similar to that of NMDA alone (Fig 2B1); in other neurons, isoproterenol pretreatment attenuated the NMDA-induced cAMP to below that of NMDA alone (Fig 2B2). Note that in all cases the NMDA after ISO response is measured as the difference between the response to isoproterenol pre-treatment and the response to the combined ISO+NMDA application. Thus, if the two responses were additive, the NMDA after ISO response would be the same as in response to NMDA alone. A synergistic effect would produce an NMDA after ISO response larger than the response to NMDA alone. Statistical analysis revealed that the average NMDA-induced cAMP response of the NMDA after ISO stimulus was significantly attenuated relative to that of NMDA alone in the neurites but not in the soma (NMDA alone, *n* = 46; NMDA after ISO, *n* = 10; neurites: P = 0.03; soma: P = 0.337; Fig 2D). In addition, we observed an inverse relationship between isoproterenol- and NMDA-induced cAMP responses in the soma, such that as the cAMP response to isoproterenol increased, that of subsequently applied NMDA decreased (Fig 2E). Since we did not observe synergistic cAMP production in these neurons, we hypothesized that additional negative feedback mechanisms were operating downstream of βARs in these neurons to limit the subsequent NMDA-induced cAMP.

### PKA and PDE4s are involved in the isoproterenol-induced reduction of NMDA-induced cAMP

**Fig 3:**
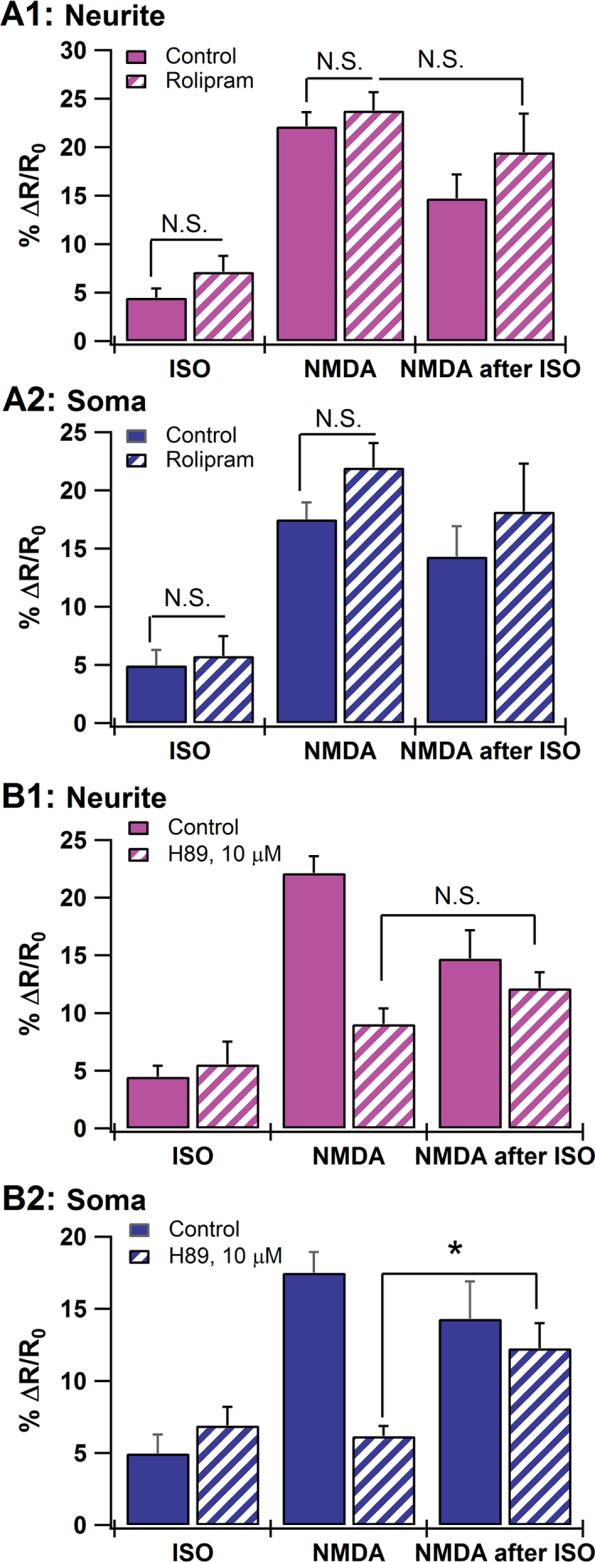
cAMP during experimental perturbation of the cAMP-PKA-PDE4 pathway in cultured hippocampal neurons. **A.** Effect of rolipram (1 μM) on the cAMP response to NMDA (*n* = 19) and the NMDA after isoproterenol stimulus (*n* = 13) in the neurite (**A1**) and soma (**A2**). Rolipram prevents the attenuation of the NMDA after ISO response observed in the neurite, but produces no significant effect in the soma. We used a subsaturating dose to focus on the effect of rolipram on the interaction between NMDA and ISO, and to prevent a large change in the NMDA alone or ISO alone cases. In addition, the subsaturating rolipram ensured we did not saturate the FRET sensor. **B.** Effect of PKA inhibition by H89 (10 μM) on the cAMP response to isoproterenol (*n* = 8), NMDA (*n* = 11), and the NMDA after ISO stimulus (*n* = 8). H89 prevents the attenuation of the NMDA after ISO response in the neurite (**B1**) and allows ISO pretreatment to enhance the soma response (**B2**). Note that, for ease of comparison with the averaged cAMP response to NMDA alone, the cAMP response to the NMDA after ISO stimulus is the difference between the peak response to NMDA after ISO and the response to the initial ISO application. All data represent the means and SEM.

Because PDE4s are the predominant negative feedback regulators of cAMP signaling in hippocampal neurons [17], we investigated their role in the isoproterenol-mediated attenuation of the NMDA response. We bath applied a subsaturating concentration (1 μM) of the specific PDE4 inhibitor rolipram prior to NMDA alone, and the NMDA after ISO stimulus. Rolipram did not increase the average cAMP response to isoproterenol or to NMDA alone in either neurites or soma (Fig 3A). Nonetheless, rolipram prevented the decrease in cAMP response caused by isoproterenol pretreatment in the neurites (rolipram + NMDA, *n* = 19; rolipram + NMDA after ISO stimulus, *n* = 13; neurites: P = 0.341; Fig 3A1). In other words, in the presence of rolipram, the peak neurite response to NMDA after ISO (%ΔR/R_0_ = 26.7) approximately equaled the sum of the isoproterenol response (%ΔR/R_0_ = 7.2) plus the NMDA response (%ΔR/R_0_ = 23.8). These data suggest that PDE4s may be involved in reducing the NMDA-induced cAMP response following isoproterenol pretreatment.

PKA phosphorylation of PDE4 can enhance its hydrolytic activity ~2-fold [18,19], acting as a negative feedback regulator of cAMP [20,21]. If this mechanism is operating in hippocampal neurons, then inhibiting PKA should prevent the attenuation of the NMDA response by prior isoproterenol application. Application of the specific PKA inhibitor H-89 (10 μM) prevented the reduction in the NMDA response caused by prior application of isoproterenol in the neurites (NMDA in H89, *n* = 11; NMDA after ISO stimulus in H-89, *n* = 8; *p* = 0.138; Fig 3B1), and allowed the ISO pretreatment to enhance the soma response to NMDA (P = 0.0095; Fig 3B2). Note that inhibition of PKA with H-89 did not alter cAMP responses to isoproterenol alone in either neurites or soma (ISO, *n* = 10; H-89 + ISO, *n* = 8; neurites: P = 0.603; soma: P = 0.315; Fig 3B) but robustly decreased NMDA-induced cAMP responses in both neurites and soma (NMDA, *n* = 46; H-89 + NMDA, *n* = 11; neurites: *P* < 0.001; soma: P < 0.001; Fig 3B). The latter is consistent with the known function of PKA phosphorylation in increasing the fractional Ca^2+^ influx through NMDARs in CA1 pyramidal neurons [73,74]. Nonetheless, in the neurite in the presence of H-89, the peak response to NMDA after ISO equaled the sum of the isoproterenol response plus the NMDA response, as observed with rolipram. Therefore, the experiments suggest that both PDE4s and PKA may be involved in the attenuation of NMDA-induced cAMP following isoproterenol pretreatment.

### The cAMP-PKA-pPDE4 negative feedback loop cannot completely explain the ISO-attenuated NMDA response

**Fig 4:**
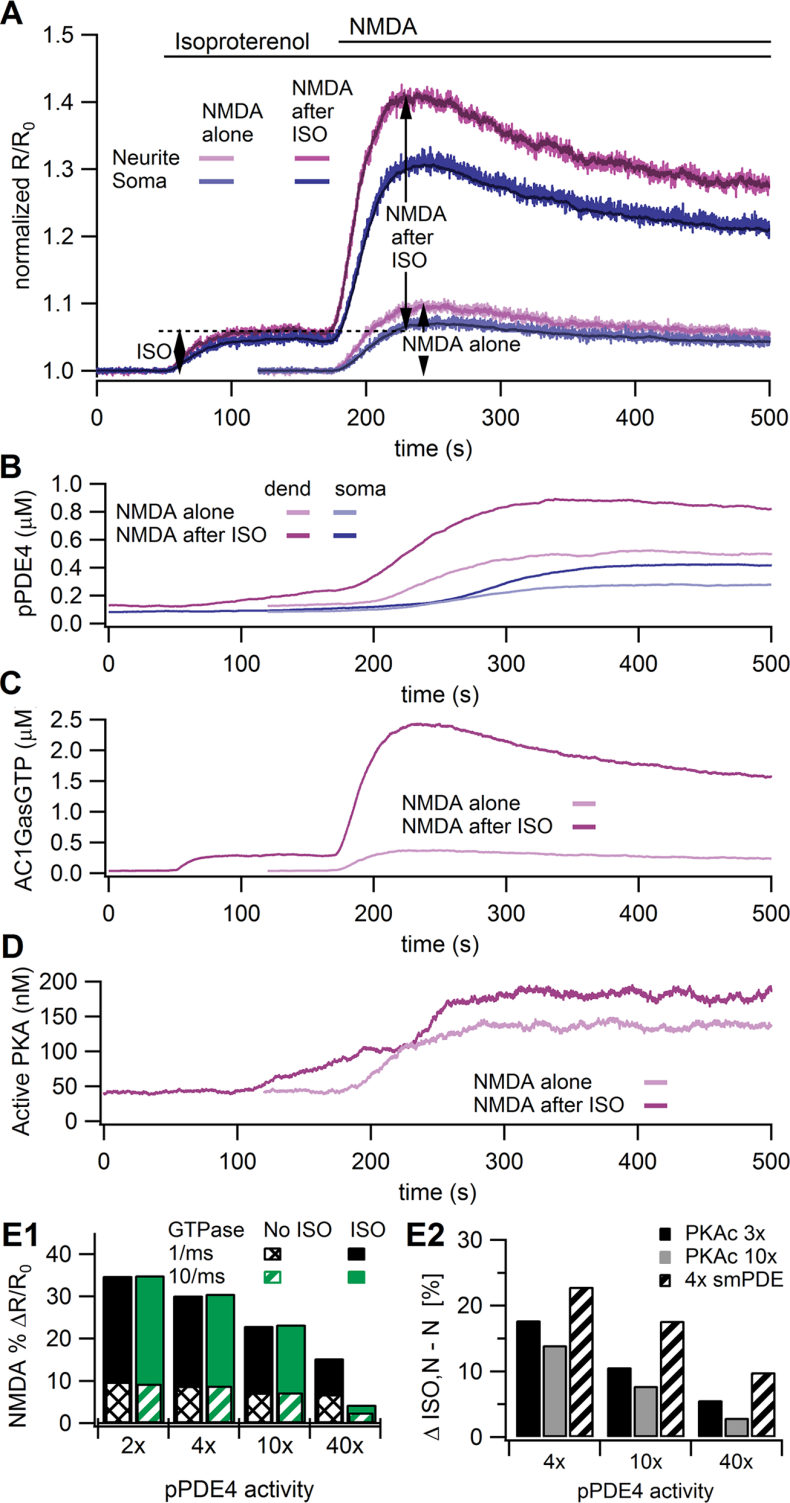
Model simulation of cAMP dynamics controlled by mechanisms downstream of AC. **A.** Traces of the cAMP responses to NMDA alone, and NMDA after ISO in the model with pPDE4 activity twice that of PDE4 activity. Arrows and dashed lines show how response amplitudes were measured, both for the model and for the experiments. Thus, the initial (ISO) part of the NMDA after ISO trace is considered ISO alone. The trace for NMDA alone has been offset 120s for ease of comparison. The NMDA alone traces and the initial (ISO) part of the NMDA after ISO traces agree with the experimentally observed cAMP response to ISO or NMDA alone, including the soma to neurite gradient. Nonetheless, the model cAMP in response to NMDA after ISO does not agree with experimental data, suggesting that some other mechanisms are operating in these cells. The darker, less noisy lines near the center of the traces show the cytosolic concentrations, which are slightly lower than the submembrane value for the soma due to a small gradient. **B.** Traces showing the dynamics of PDE4 phosphorylation in response to NMDA after ISO. The maximal phosphorylation is reached in ~3 minutes, which is slightly faster than the ~6 minutes reported for PDE4D5 in [19]. The purpose of the slight increase in rate of phosphorylation was to enhance the operation of the negative feedback loop. Note that pPDE4 increases for both NMDA alone and NMDA after ISO. **C.** Traces showing that GsαGTP bound adenylyl cyclase increases tremendously after ISO, leading to dramatically increased cAMP production. **D.** Traces showing that PKA activity is only moderately higher after ISO than with NMDA alone, explaining the modest increase in pPDE4 with ISO compared to NMDA alone. **E.** Effect of parameter variations on dendritic cAMP response. **E1.** Increases in pPDE4 activity and GsαGTP activity were not sufficient to reproduce experimental results. Solid bars show response to NMDA after ISO, striped or hatched bars show response to NMDA alone. Similar to experiments, the cAMP response to the NMDA after ISO stimulus is the difference between the peak response to NMDA after ISO and the response to the initial ISO application measured just prior to NMDA application. Though 40x lowered the NMDA response after ISO, the NMDA response without ISO was also reduced to a value below that observed experimentally. **E2.** Increases in the rate of PKA phosphorylation of PDE4 reduces the difference between NMDA after ISO and NMDA alone, but is not sufficient to make the NMDA after ISO response smaller than the NMDA alone response. ΔISO,N-N is the difference between the NMDA after ISO response and the NMDA alone response.

To better understand how isoproterenol-induced enhancement in PDE4 activity might lead to an attenuation of the cAMP response to NMDA, we adapted a previously validated, spatial mechanistic model of signaling pathways in CA1 pyramidal neurons [75] and evaluated whether downstream mechanisms alone, i.e., the cAMP-PKA-pPDE4 negative feedback loop (Fig 1A), can indeed account for the experimental observations. We ran the same stimulation combinations of NMDA with and without isoproterenol pretreatment. Using this initial model we verified in control simulations that the cAMP responses to isoproterenol alone (Fig 4A—initial part of NMDA after ISO trace) and NMDA alone (Fig 4A) agreed with the experiments. Indeed, both the dynamics and the existence of a soma to neurite gradient were similar to experiments. The fluctuations in the model traces are due to the stochastic nature of the molecule interactions in the small submembrane region. The standard deviation of these signals ranges from 0.4 to 0.8%ΔR/R_0_. The darker, less noisy, superimposed trace shows the concentration in the cytosolic compartments. Because the mean values are similar for cytosolic and submembrane traces, only the less noisy cytosolic traces are shown in the remainder of the graphs. In addition to similarity in cAMP responses, the time course of pPDE4 activity (Fig 4B) was consistent with that shown by others [19]. Note that the lag in phosphorylation of PDE4 in the soma is due to the lower surface to volume ratio in the soma. The adenylyl cyclase is in the membrane, whereas the PDE4s are throughout the morphology; this higher adenylyl cyclase to PDE4 ratio in the neurite causes a higher neurite cAMP and PKA activity, and faster phosphorylation of the PDE4s in the neurite.

We tested the hypothesis that the cAMP-PKA-PDE4 negative feedback loop was involved in the attenuation by evaluating the NMDA after ISO stimulus in the model. However, the NMDA-induced cAMP response following isoproterenol was synergistic, not attenuated, relative to the cAMP response to NMDA alone (Fig 4A). In other words, the difference between the peak NMDA after ISO response and the isoproterenol response (%ΔR/R_0_ = 34.9) was greater than the NMDA alone response (%ΔR/R_0_ = 9.8); also, the peak NMDA after ISO response (%ΔR/R_0_ = 40.9) was greater than the sum of the NMDA alone and ISO alone responses (%ΔR/R_0_ = 16.0). The cause of the synergistic response to NMDA after ISO was the greatly increased cAMP production by the GsαGTP bound adenylyl cyclase (Fig 4C), which is not sufficiently compensated by the increase in pPDE4, because PKA and pPDE4 also increase in response to NMDA alone (Fig 4B and 4D).

We further evaluated whether the cAMP-PKA-PDE4 negative feedback loop could explain the experimentally observed attenuation of the NMDA response after ISO by assessing two other mechanisms that could enhance PDE4 activity in an activity-dependent manner. First, to see if an enhanced activity of pPDE4 could underlie the attenuation, we simulated the effect of a several-fold increase in pPDE4 activity, as may occur due to SUMOylation [76]. Increasing the activity of pPDE4 did not eliminate the enhanced response to NMDA after ISO application, because enhanced pPDE4 activity also decreased the NMDA alone response (Fig 4E1), even when combined with an increased rate of GsαGTP hydrolysis (Fig 4E1). Combining enhanced pPDE4 activity with an increase in the rate at which PKA phosphorylates PDE4 reduced, but did not eliminate, the enhanced response to NMDA after ISO application (Fig 4E2). Since previous work has shown that PDE4s are anchored [26], and that different PDE4 isoforms distribute differentially in cells [77], we repeated these simulations with four times the concentration of submembrane PDE4 compared to cytosolic PDE4; however, the cAMP response to the NMDA after ISO stimulus remained larger than that to NMDA alone (Fig 4E2), indicating that the mechanisms integrated in this initial model were not sufficient to reflect the responsible pathways.

**Fig 5:**
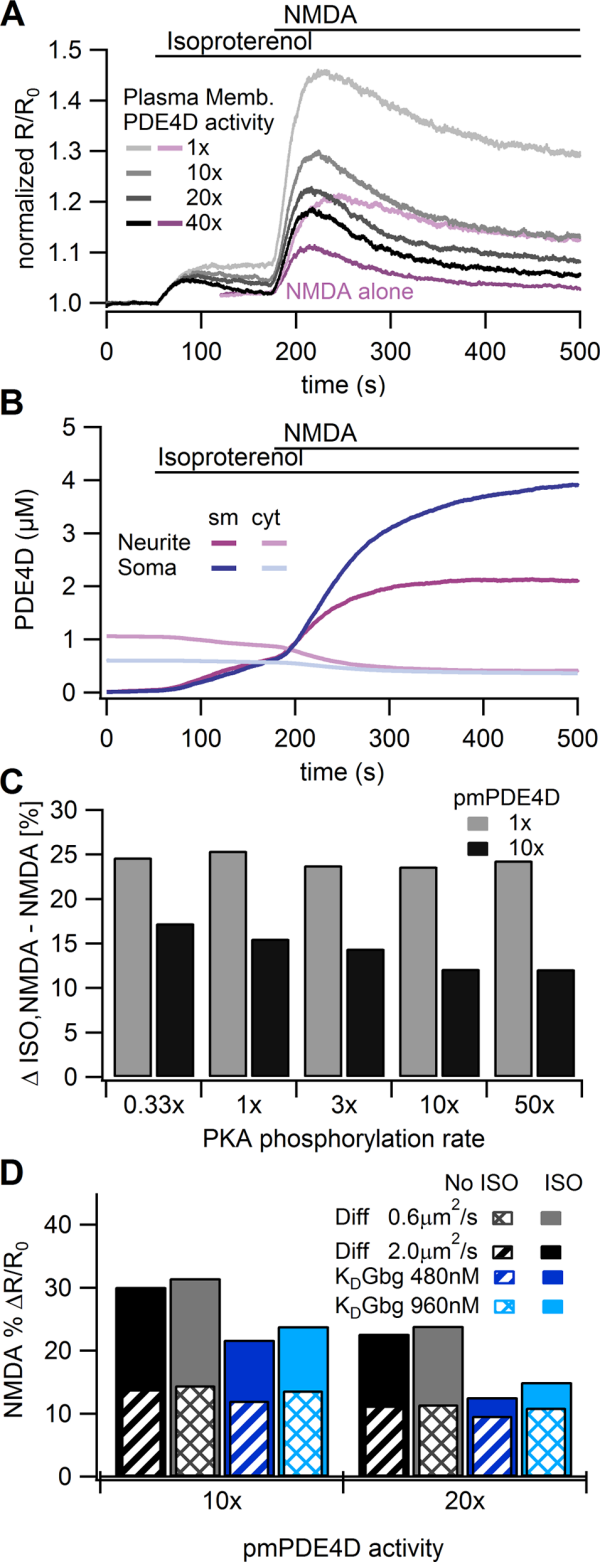
Model variations in PDE4 cannot produce a reduced cAMP response to NMDA after isoproterenol pretreatment. **A.** Traces showing effect of dynamic recruitment of PDE4D on cAMP response in model neurites, with four activity rates for plasma membrane PDE4D. For the 10x case, the total NMDA after ISO (%ΔR/R_0_ = 29.0) is greater than the sum of the NMDA (%ΔR/R_0_ = 9.4) and ISO (%ΔR/R_0_ = 5.9) responses. Standard deviation of these cytosolic trace ranged from 0.2%ΔR/R_0_ to 0.6%ΔR/R_0_; standard deviation of the submembrane traces (not shown) ranges from 0.4%ΔR/R_0_ to 1.0%ΔR/R_0_. The NMDA alone cases are shown for 1x and 40x plasma membrane PDE4D activity. The reduction in the NMDA alone case with 40x plasma membrane PDE4D prevents the enhanced PDE4D activity from reproducing the experimental results. **B.** Traces showing the concentration of total PDE4D in the membrane and cytosol during dynamic recruitment to show that stimulation by isoproterenol causes an increase of PDE4D in the membrane and a decrease in the cytosol. **C.** Difference in peak Fret between NMDA after ISO and NMDA alone (ΔISO,NMDA-NMDA) for a range of PKA phosphorylation rates and plasma membrane (pm) PDE4D activity. **D.** Neither variations in diffusion constant nor a change to Gβγ dependent recruitment of PDE4D can reproduce the experimental results. Solid bars show response to NMDA after ISO, striped or hatched bars show response to NMDA alone, which decrease as pmPDE4D activity is increased. Similar to experiments, the cAMP response to the NMDA after ISO stimulus is the difference between the peak response to NMDA after ISO and the response to the initial ISO application measured just prior to NMDA application.

A second mechanism of enhancing apparent PDE4 activity is via dynamic recruitment of the PDE4D5 subtype to the plasma membrane [26,78,79]. Bringing PDE4D5 in close proximity to adenylyl cyclases following βAR activation can increase the specificity and efficiency of PDE4D5 activity and could thus strongly oppose subsequent cAMP generation at the plasma membrane. To test if this could explain the attenuation, we implemented dynamic recruitment of PDE4s in the model (see Methods; Table 4) such that a fraction of the PDE4 (called PDE4D in our model) was recruited to the submembrane region following isoproterenol pretreatment. The activity of the membrane-bound PDE4D was either the same as the cytosolic PDE4D, or increased, as may occur when PDE4D binds anchoring proteins at the submembrane [80]. Fig 5B shows an increase in PDE4D in the submembrane with stimulation, demonstrating that the simulated dynamic recruitment is indeed successful. However, the NMDA-induced cAMP response following isoproterenol was still much greater than that of NMDA alone, even with enhanced activity of the membrane-bound PDE4D (Fig 5A). As observed with the basic model, enhanced plasma membrane PDE4D activity does not reproduce the experimental results because the enhanced plasma membrane PDE4D reduces the NMDA alone response, though much less than observed with the basic model (Fig 5A and 5D). Because dynamic recruitment of PDE4D with enhanced membrane-bound PDE4D activity seemed promising, we performed additional simulations of this model variant with faster or slower rates at which PKA phosphorylates PDE4 and different rates at which PDE4D diffuses from the cytosol to the membrane. Increasing the rate at which PKA phosphorylated PDE4 indeed reduced the NMDA response after ISO, though not enough to account for the experimental observations, whether with or without enhanced activity of plasma membrane PDE4 (Fig 5C). Slowing the diffusion constant of PDE4D (Fig 5D) had only a small effect, because of the small diameter of the dendrite. Similar to the cAMP-dependent recruitment of PDE4D, Gβγ-dependent recruitment of PDE4D (Fig 5D) was unable to lower the cAMP response to NMDA to a value lower than the cAMP response to NMDA after ISO. Thus, we conclude that the negative feedback loop of PKA phosphorylation of PDE4, while effective, is insufficient to fully explain the suppression of synergistic cAMP generation induced by NMDA after ISO in hippocampal neurons.

### PKA-mediated Gs-Gi switching regulates isoproterenol-induced attenuation of NMDA-induced cAMP

As an alternative to mechanisms acting downstream of adenylyl cyclases in the cAMP signaling network, we considered mechanisms operating upstream of adenylyl cyclases to regulate cAMP. One possibility is regulation of βARs, which are known to undergo two modes of desensitization, one mediated by PKA and another by G protein-coupled receptor kinases (GRK) [81]. In particular, PKA phosphorylation of βARs can lead to a “switch” in βAR coupling from the Gs to Gi subtype of GTP binding proteins [62,82]. Activated Gi proteins can then release GiαGTP and Giβγ subunits that can directly inhibit the catalytic activity of AC1 [70,83], thus reducing cAMP production. However, the effects of PKA-mediated desensitization of βARs on NMDA-induced cAMP have yet to be investigated.

To evaluate if PKA-mediated desensitization of βARs could explain the attenuation of NMDA-induced cAMP following isoproterenol pretreatment, we added PKA-dependent Gs-Gi switching and GiαGTP inhibition of AC1 to the model (see Materials and Methods; Fig 1C; Table 5). We implemented PKA phosphorylation of βARs on four serine residues, as these have been identified as PKA phosphorylation sites *in vitro* [67]. Fully phosphorylated βARs bind the Gi subtype of G protein instead of the Gs subtype, producing GiαGTP, which can then bind to and inhibit AC1.

**Fig 6:**
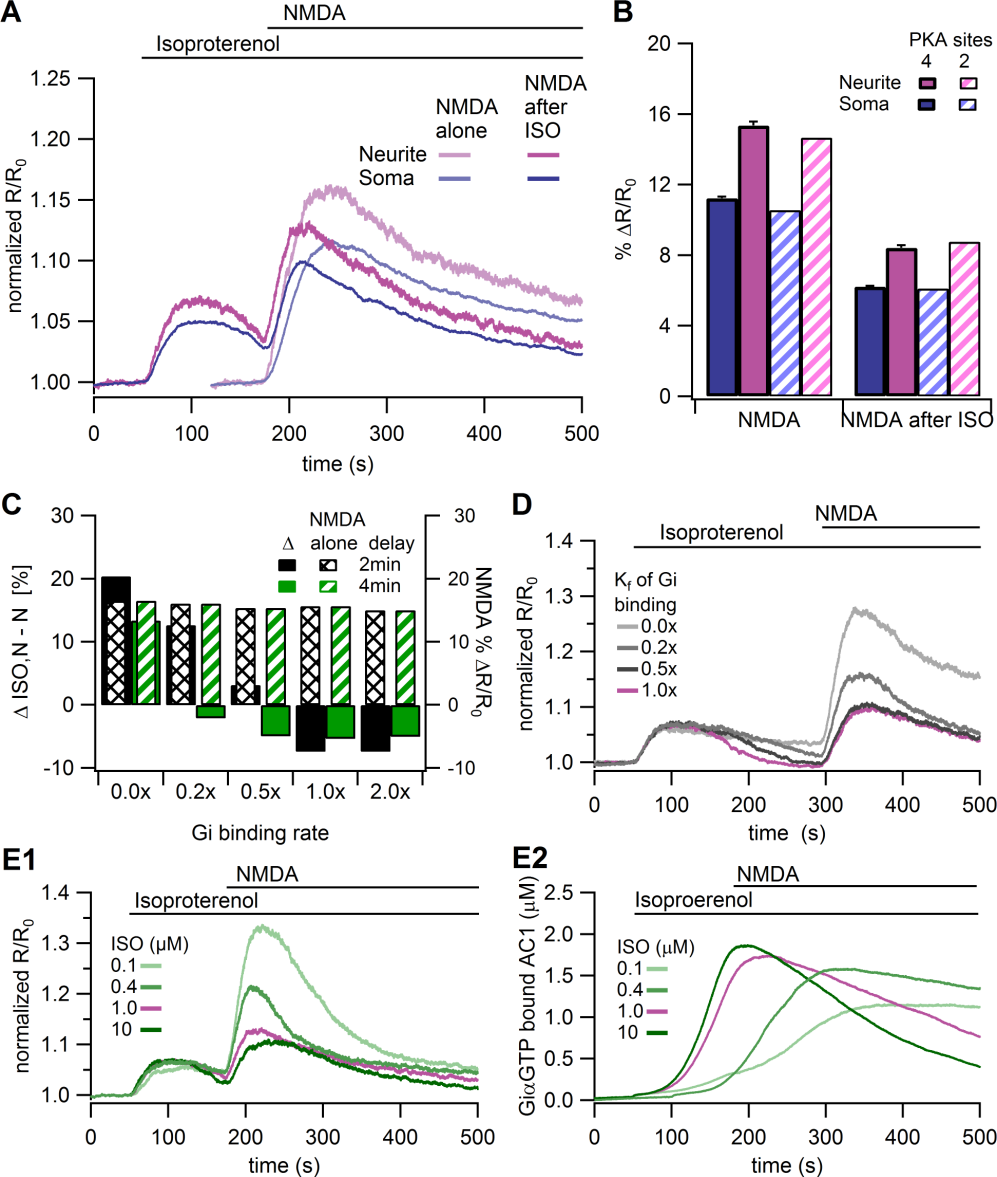
Effects of Gs-Gi switching and GiαGTP inhibition of AC1 in the model. **A.** Traces of the cAMP responses to NMDA alone and NMDA after ISO in the presence of Gs-Gi switching and GiαGTP inhibition of AC1. In the soma, the total NMDA after ISO response of (%ΔR/R_0_ = 9.6) is slightly less than the sum of the NMDA (%ΔR/R_0_ = 11.4) + ISO (%ΔR/R_0_ = 4.9) responses. Similarly in the dendrite, the total NMDA after ISO (%ΔR/R_0_ = 12.5) is much less than the sum of the NMDA (%ΔR/R_0_ = 15.6) and ISO (%ΔR/R_0_ = 6.6) responses. Standard deviation of these cytosolic traces range from 0.2%ΔR/R_0_ to 0.4% ΔR/R_0_; standard deviation of the submembrane traces (not shown) is approximately twice that of the cytosolic traces. **B.** Amplitude of cAMP response for the conditions in A (4 PKA phosphorylation sites on βAR; mean and SEM, *n* = 3) and also in a model with only 2 PKA phosphorylation sites on the βAR. **C.** Summary of the effect of Gi binding rates on the difference between the NMDA after ISO response and the NMDA alone response (ΔISO,N-N; Solid bars). Model neurite cAMP response to NMDA after ISO is smaller than the response to NMDA alone for Gi binding rates to βARs between 0.5x and 2.0x of control. The Gi binding rate had no effect on the NMDA alone response (striped or hatched bars). **D.** Neurite cAMP response when NMDA is applied 4 min after isoproterenol exhibits attenuation of NMDA response, with smaller cAMP response decay when rate of Gi binding is lower (0,2x), but not without Gi binding (0.0x). **E.** Differential cAMP response due to various ISO concentrations in model neurites. **F.** The amplitude and time course of inhibited AC1 in model neurites in response to different ISO concentrations reveals that larger ISO produces a smaller NMDA response due to Gi inhibition of the Gs-bound AC1.

Simulations demonstrate that switching can explain the reduction in NMDA induced cAMP following isoproterenol application. In control simulations, we observed that there was little difference in isoproterenol- or NMDA-induced cAMP (Fig 6A) with the addition of Gs-Gi switching and GiαGTP inhibition of AC1; however, when we simulated the NMDA after ISO stimulus, Gi robustly inhibited the NMDA-induced cAMP increase (Fig 6A and 6B). After isoproterenol pretreatment, the elevation in cAMP produced by NMDA stimulation was similar to that observed experimentally and smaller than to NMDA alone. This response was robust to parameter variations, as similar results were obtained with a model where PKA phosphorylation of βARs could occur only on two sites (Fig 6B). In addition, the attenuation of the NMDA response after ISO was observed for a range of affinities of Gi for the phosphorylated receptor (Fig 6C). Gs-Gi switching differs qualitatively from enhanced PDE4 activity in that switching only occurs consequent to the ISO application and does not affect the NMDA alone response (Fig 6C). Simulations of a 4 min delay between NMDA and ISO application (Fig 6D) produces too strong a decay of the ISO response using the default parameters; however a lower Gi binding rate, e.g. 0.2x, yields a much smaller decay of the ISO response while still attenuating the subsequent NMDA response. The attenuation of the NMDA response after ISO also was observed for a range of isoproterenol concentrations (Fig 6E1), though the NMDA response after ISO increased with lower concentrations of isoproterenol. The amount of GiαGTP bound to AC1 in response to different isoproterenol concentrations reveals the mechanism underlying this observation. The time course and strength of inhibition of AC1 by is proportional to the concentration of isoproterenol (Fig 6E2), and a fast increase in GiαGTP is needed to inhibits the subsequent peak cAMP response to NMDA.

**Fig 7:**
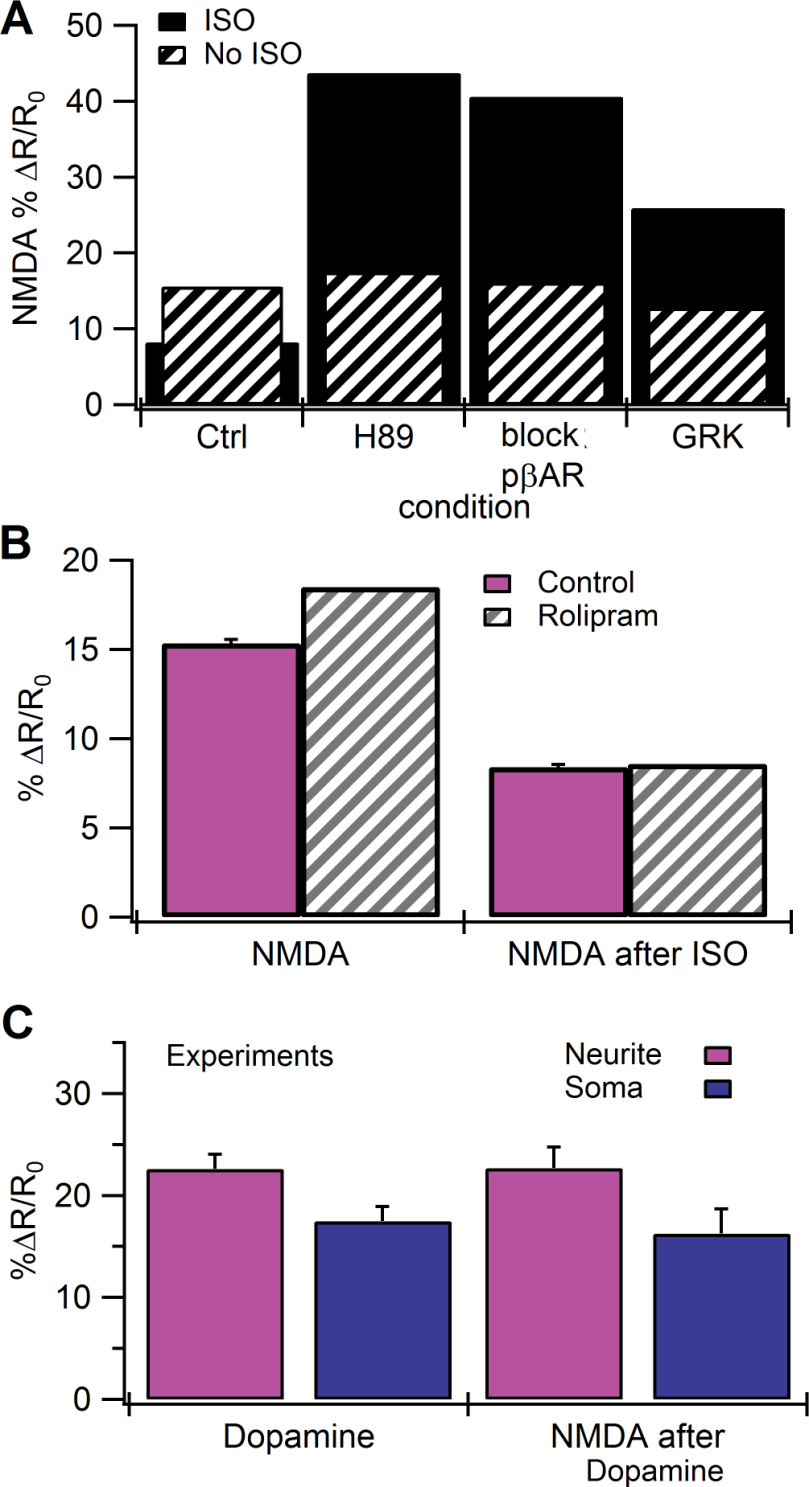
Role of PKA phosphorylation of βAR in the model. **A.** In the model neurites, minimal attenuation of the cAMP response to the NMDA after ISO stimulus is observed in the absence of PKA phosphorylation of βARs (block pβAR), or when all PKA forms are blocked (H89). Desensitization of βARs by GRK (without Gi inhibition of AC1) does not produce the experimentally observed decrease in the cAMP response to NMDA after ISO pretreatment. **B.** Simulations of rolipram do not eliminate the reduction in NMDA response after isoproterenol application. ***C***. Amplitude (mean and SEM) of cAMP responses show no attenuation to NMDA after dopamine (*n* = 11) in cultured hippocampal neurons.

To further explore the mechanisms involved in producing the observed results we performed several additional simulations. To evaluate the specific roles of PKA in the model, we either blocked PKA phosphorylation of βAR or blocked all PKA activity. Either blocking PKA phosphorylation of βARs or inhibiting total PKA activity (e.g. with H89) prevented the attenuation of the NMDA-induced cAMP following isoproterenol (Fig 7A). This latter result, that inhibiting total PKA activity blocks the attenuation of the cAMP response caused by isoproterenol pretreatment, is similar to experiments using the PKA inhibitor H89. The difference between inhibiting total PKA and preventing PKA phosphorylation of βARs shows the contribution of pPDE4 (with the 2x increase in activity) to reducing the cAMP response to NMDA after isoproterenol.

Two components of the PKA-mediated desensitization of βARs could be producing the observed attenuation of the NMDA response after ISO. First, decoupling of βAR from Gs may remove the synergistic activation of AC1 by reducing the production of GsαGTP, independent of the inhibition of AC1 by GiαGTP. Second, the direct inhibition of AC1 by GiαGTP may reduce AC1 activity to a level below that of NMDA alone. Simulations in a model in which GiαGTP did not bind to the phosphorylated βAR (Fig 6C, Gi bind rate = 0.0x) allow the NMDA response after ISO to exceed the NMDA alone response, demonstrating that decoupling the receptor from Gs to Gi by itself is not sufficient. An alternative method for evaluating the role of Gs decoupling is to replace PKA phosphorylation of βAR with GRK mediated desensitization, which is responsible for the transience of the isoproterenol-induced cAMP response in HEK293 cells [84,85]. GRK-mediated desensitization of β_2_ARs leads to the recruitment of β-arrestin to the receptor, which is required for receptor desensitization via internalization, recycling, and degradation [86]. To see if GRK-mediated desensitization of βARs could explain the attenuation of the cAMP response to NMDA after ISO, we implemented GRK-mediated desensitization of βARs in the model in the absence of Gs-Gi switching (Table 6), together with dynamic recruitment of PDE4D to the membrane (and 10x enhanced activity of plasma membrane PDE4D). The GRK-mediated desensitization produced a response to NMDA after ISO (Fig 7A) that was larger than the response to NMDA alone, and was unable to reproduce the experimentally observed (Fig 2D) attenuation of the NMDA response after isoproterenol pretreatment. In summary, desensitization of the βAR decreased the synergistic activation of AC1, but did not reduce the cAMP response to NMDA after ISO to a level 25% less than that of NMDA alone. Thus, in addition to receptor decoupling from Gs, inhibition of AC1 by GiαGTP is required.

**Table 6:** Reactions and rate constants for GRK-mediated desensitization in the model.

Reaction	kf (nM^-1^ ms^-1^)	kb (ms^-1^)	source
Iso-R ↔ Iso-Rdesens	2.30E-05	5.00E-07	[87]
Iso-R-Gs ↔ Iso-Rdesens-Gs	2.30E-05	5.00E-07	[87]

Though the switching model agrees with experiments regarding a role of protein kinase A, the model is unable to reproduce the experimental observation that rolipram prevents the reduction in the NMDA response after isoproterenol pretreatment. Rolipram in the model was implemented as inhibition of a fraction of the PDE4, both because the affinity of rolipram for PDE4 depends on the isoform and to reproduce the experimental observation that rolipram causes only a small increase in cAMP basal level. Simulations show that rolipram slightly enhances the cAMP response to either isoproterenol, or to NMDA alone (Fig 7B), similar to experiments. The consequence of enhanced cAMP in response to ISO is a slightly enhanced PKA phosphorylation of the remaining PDE4, leading to a similar or slightly reduced NMDA response, which is opposite of that shown by experiments. The same result occurs whether rolipram inhibits 5% or 10% of the PDE4. Nonetheless, we cannot rule out that inhibition of a particular nanodomain of PDE4 in the model would be able to produce the experimental observations.

We propose that PKA-mediated Gs-to-Gi switching of βARs and GiαGTP inhibition of AC1 might underlie the reduction in the NMDA-induced cAMP response following isoproterenol pretreatment. This result implies that attenuation of the NMDA response will be blocked by the Gi inhibitor pertussis toxin [62] and that the attenuation of the NMDA response will not be observed subsequent to stimulation of Gs coupled receptors that do not exhibit switching. This latter model prediction is consistent with the results of additional experiments in which we used dopamine instead of isoproterenol to stimulate Gs coupled dopamine D1/D5 receptors in hippocampal cultures. In these experiments, there was no evidence that dopamine attenuated the subsequent cAMP response to NMDA (Fig 7C).

Additional simulations were performed to explore the implications of the model and make additional, experimentally-testable predictions. Because switching occurs only in response to isoproterenol application, and does not occur in response to NMDA application, the order and timing of agonist application will influence the cAMP response. Thus, we performed simulations with NMDA applied either prior to or simultaneously with isoproterenol application. In addition, we applied pairs of transient stimulation pulses of the same agonist.

**Fig 8:**
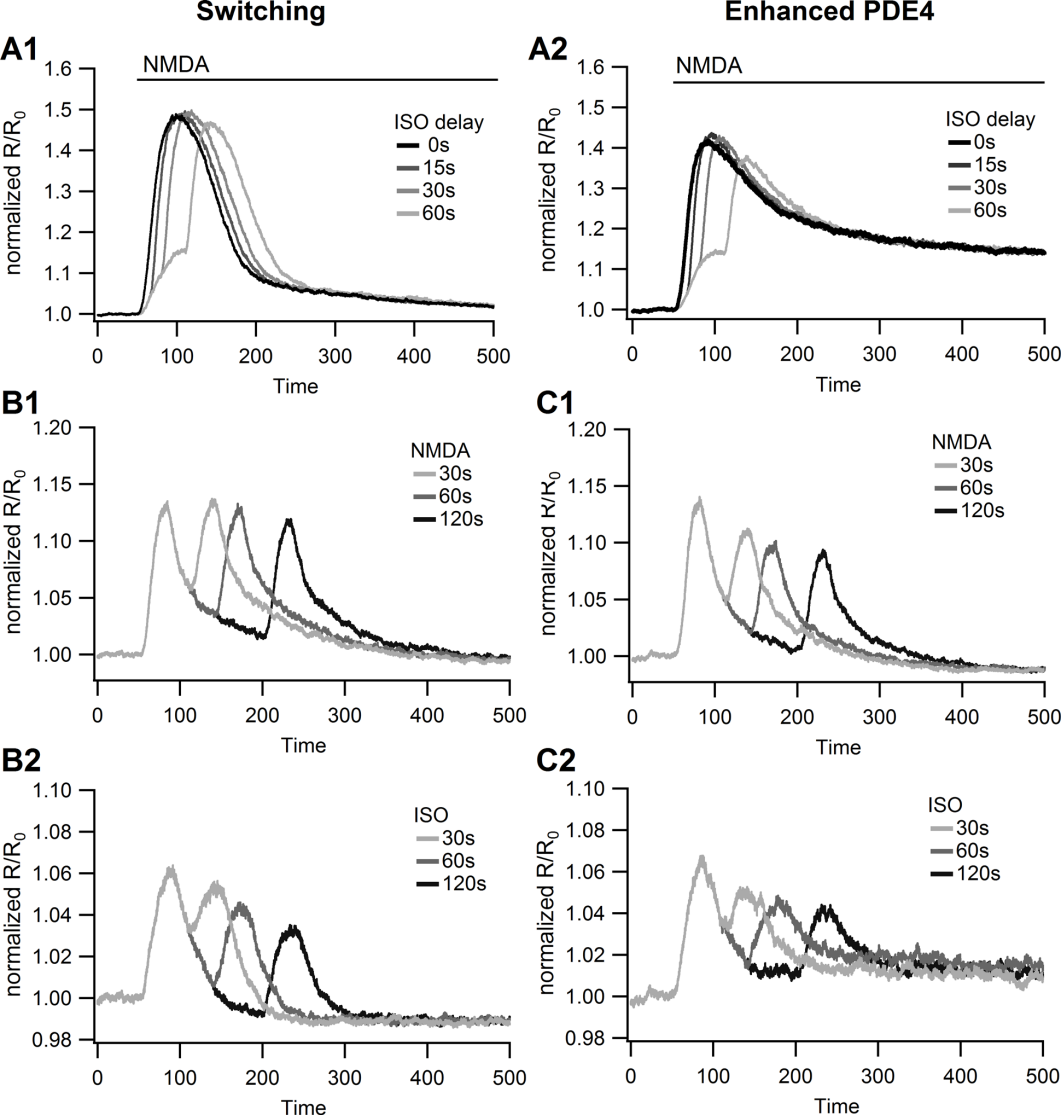
Model response to different temporal patterns of stimulation. **A.** Response to NMDA applied simultaneously with or prior to isoproterenol (ISO). Both the switching model (**A1**), and the best model without switching (**A2**: dynamic recruitment of PDE4s to the membrane, 10x activity of plasma membrane PDE4) predict a synergistic response to NMDA followed by ISO. **B.** Response of switching model to paired 30 sec pulses of NMDA (B1) or isoproterenol (B2) separated by 30, 60 or 120 sec. The switching model exhibits no decrease in the response to the second NMDA pulse compared to the first, whereas it exhibits a decrease in the response to the second isoproterenol pulse. ***C***. Response of model with enhanced PDE4 to paired 30 sec pulses of NMDA (C1) or isoproterenol (C2) separated by 30, 60 or 120 sec. The enhanced PDE4 model exhibits a decrease in the response to the second pulse of both NMDA and isoproternol with longer time delays.

Fig 8A1 shows that application of isoproterenol simultaneous with (or after) the NMDA application produces a synergistic cAMP response. The peak response of 47.7% ΔR/R_0_ was considerably greater than the sum of the NMDA alone (%ΔR/R_0_ = 15.4) and isoproterenol alone (%ΔR/R_0_ = 6.6) responses. Even the model with enhanced PDE4 (the model with dynamic recruitment and 10x activity of plasma membrane PDE4D from Fig 5A1) exhibits a larger response when NMDA is applied simultaneous with or 15–30s prior to isoproterenol (Fig 8A2). This synergy is caused by enhanced activity of AC1 when bound to both Gs and calcium-calmodulin. Because both models produce similar peak responses under these conditions, an experiment with simultaneous application of the two agonists will neither support nor refute the switching model, and instead will test a critical underlying assumption of the model: that a single pool of AC1 responds to both NMDA and isoproterenol. Simulations with a delay between NMDA and ISO application do reveal a difference: a 60s delay yields a reduced response to ISO in the enhanced PDE4 model, but not the switching model. This reduction is due to prior NMDA producing enhanced PDE4 through cAMP and PKA activity.

The response to paired pulses is another experiment that can validate the switching model. Fig 8B and 8C shows the response to the paired pulse protocol, for both the switching model (Fig 8B) and the enhanced PDE4 model (Fig 8C). Both models give a similar response to paired isoproterenol pulses (Fig 8B2 and 8C2): responses to subsequent pulses of isoproterenol are reduced with larger time intervals. The two models differ in their response to NMDA (Fig 8B1 and 8C1): the enhanced PDE4 model exhibits a significant reduction in the response to the second NMDA pulse as the time between pulses is increased, because both ISO and NMDA produce the cAMP that leads PDE4D recruitment to the membrane. The reduction does not occur in the switching model because NMDA does not produce receptor decoupling. In summary, the response to paired isoproterenol pulses will be different than the response to paired NMDA pulses in the switching model, but not in the enhanced PDE4 model, because both NMDA and isoproterenol activate PKA and enhance PDE4D equally.

## Discussion

In this work, we used a combination of FRET imaging of cAMP dynamics and spatial mechanistic modeling of cAMP signaling pathways to investigate the contribution of βAR signaling pathways to cAMP dynamics. We demonstrated that isoproterenol pretreatment of cultured hippocampal neurons leads to a reduced cAMP response to NMDA application. This result was unanticipated because AC1 is synergistically activated by Ca^2+^ and isoproterenol when applied simultaneously, as measured by cAMP in HEK293 cells [16], or cAMP-mediated transcription in cultured hippocampal neurons [15]. We showed that mechanisms both upstream and downstream of adenylyl cyclase oppose the synergistic activation of AC1 and contribute to the observed reduction in NMDA-induced cAMP caused by prior isoproterenol application. Downstream of adenylyl cyclase, the cAMP-PKA-PDE4 negative feedback loop contributes modestly to the attenuation of NMDA-induced cAMP after isoproterenol. Upstream of adenylyl cyclase, PKA phosphorylation of βARs followed by Gs-Gi switching and GiαGTP inhibition of AC1 is required to overcome the enhanced cAMP production by adenylyl cyclase stimulated by GsαGTP and Ca^2+^/calmodulin. These mechanisms are qualitatively different in that the downstream, PDE4 feedback loop is activated by both NMDA and isoproterenol, whereas the upstream βAR feedback loop is activated only by isoproterenol. Therefore, the upstream feedback loop suppresses the NMDA response after isoproterenol, but not the NMDA alone response. While in the model we have assumed GiαGTP is the G-protein subunit responsible for blocking AC1, the results do not preclude Giβγ inhibition of AC1. Indeed, the latter has been suggested to be a more potent inhibitor than the former [70,83]. Regardless of which Gi subunit is involved, both upstream and downstream mechanisms are critically dependent on PKA activity.

Both experiments and simulations revealed a gradient of cAMP from the neurites to the soma. The gradient was observed after NMDA alone, but was reduced or absent in response to NMDA after ISO application. These observed gradients are consistent with those previously reported [88], despite being measured on a smaller spatial scale. In that study, gradients were measured over a spatial scale of 100 μm for both simulations and experiments, whereas our gradients appear across a 20 μm long structure. The source of the gradients in both studies is the larger surface to volume ratio of the neurites (dendrites) as compared to the soma: the membrane location of adenylyl cyclase versus PDE4s located in the entire volume produces a greater ratio of production to degradation for neurites (dendrites) as compared to the soma. The spatial aspect of the model also contributed to the delay in dynamic recruitment of PDE4D from the cytosol to the submembrane region. Though dynamic recruitment could not completely reproduce the experimental results, it did indeed produce a small reduction in the NMDA response after isoproterenol application.

Amongst the preponderance of PKA substrates in hippocampal neurons, our results suggest that PKA phosphorylation of βARs is crucial for mediating the attenuation of NMDA-induced cAMP by isoproterenol pretreatment. PKA phosphorylates a number of different targets in CA1 pyramidal neurons which are implicated in plasticity [89]; however, the phosphorylation of the majority of these PKA targets leads to activity that would presumably promote rather than attenuate cAMP generation. For example, PKA phosphorylation of NMDARs [73] or L-type voltage-gated Ca^2+^ channels [90] increases Ca^2+^ influx through these channels, which then enhances the activation of Ca^2+^/calmodulin-stimulated adenylyl cyclases. Therefore, a robust mechanism for the reduction of cAMP is needed to overcome this array of PKA effects. There are comparatively few known PKA targets that lead to reduced cAMP signaling in CA1 pyramidal neurons. One such mechanism is PKA inhibition of AC8 [91]; however, the modest inhibition of AC8 by PKA observed in HEK293 cells (~30% reduction of FRET after 3 min forskolin stimulation) is likely too weak to reduce NMDA-induced cAMP under our conditions. This is compounded by the relatively small contribution of AC8 to the cAMP response in the first place, due to its relatively low affinity for Ca^2+^/calmodulin (~800 nM vs. ~150 nM for AC1). Nonetheless, our results cannot preclude the possibility that PKA phosphorylation of both AC1 and AC8 contributes to the experimental observations.

The negative feedback loop of PKA phosphorylation of PDE4s cannot completely account for the experimental results in part because this mechanism leads to reduction in the NMDA alone response, and in part because this limits PKA activity itself, and thus limits the amount of pPDE4. Indeed, for a negative feedback loop to allow a large but transient response (required to uphold the isoproterenol response and repress the subsequent NMDA response), a time delay followed by rapid activation of the negative feedback loop is required [92–94]. The addition of dynamic recruitment to the model produced a moderate time delay, but PDE4 enhancement still began during the isoproterenol and NMDA alone pulses. Several mechanisms, including further enhancement of pPDE4 activity by SUMOylation [76], may produce the requisite delay. Though we included the effect of SUMOylation, by allowing up to 40 fold increase in activity of plasma membrane PDE4, this effect was instant. In contrast, a delay in activation of SUMO may have been able to produce the experimental results. An alternative to SUMOylation is proffered by a recent study showing that CaMKII phosphorylation of PDE4 increases its activity in cardiac myocytes [95]. This suggests that a 10–20 fold increase in PDE4 activity of dual PKA/CaMKII phosphorylated PDE4 might provide a delay in PDE4 enhancement tied to the NMDA delay. Another possible mechanism involves the ultrasensitive switch dynamics of ERK activation [65,66]. Delayed phosphorylation of PDE4 by ERK accompanied by a large enhancement in PDE4 activity would provide the needed delay in PDE4 enhancement. ERK indeed phosphorylates some PDE4 isoforms [96], though the most common result is inhibition [97]. ERK itself could be activated via PKA phosphorylation of the βAR followed by either switching or arrestin recruitment [61], via PKA phosphorylation of B-Raf [98], or through other pathways not involving PKA. If ERK is involved in producing the experimental results, then MEK inhibitors should block the smaller NMDA response after ISO, and biochemical assays could be employed to demonstrate both an increase in ERK phosphorylation and the reduction in phosphorylated ERK when PKA inhibitors are applied.

Though the model implements the spatial detail of submembrane location for membrane bound molecules, nanodomain mechanisms may be operating in the experiments that were omitted from the model. One nanodomain mechanism involves more specific localization of PDE4 subtypes, and extrapolates from the known differential affinity of rolipram for different PDE4 subtypes [99]. This mechanism assumes that PKA phosphorylation of PDE4s is limited to those anchored in a nanodomain around the NMDA receptor, and that rolipram specifically inhibits that NMDA-associated-subset of PDE4s. Such a nanodomain of PKA phosphorylated PDE4s might yield a model response to rolipram similar to that of experiments. Another nanodomain involves localization of different pools of adenylyl cyclases. If the pool of adenylyl cyclase activated by isoproterenol were distinct from the pool of adenylyl cyclase activated by NMDA, there would be no synergistic activation of AC1 by isoproterenol and NMDA. The existence of adenylyl cyclase nanodomains could be tested experimentally: In the absence of such nanodomains, the model predicts that NMDA application simultaneous with ISO application would produce a synergistic increase in cAMP. If experiments reveal an absence in synergy, then either the dominant subtype of adenylyl cyclase or the spatial location of adenylyl cyclases in the model needs modification. Furthermore, in the absence of synergy, the enhanced PDE4 activity provided by PKA phosphorylation (Fig 4E) might be sufficient to reduce NMDA induced cAMP production.

Since the discovery of Gs-Gi switching after PKA phosphorylation of β-adrenergic receptors, the signaling pathways downstream of switching have been characterized in several cell types [62,63,100,101], but have only recently been considered in neurons. Prior research on HEK293 cells showed that ERK activation was dependent both on PKA activity and on pertussis toxin sensitive G proteins [62]. In CHO cells, norepinephrine-stimulated ERK activation specifically requires PKA phosphorylation of β-adrenergic receptors [63]. In the hippocampus, recent experiments [102] employing novel βAR antagonists suggest that switching is involved in the LTP underlying memory storage. Specifically, theta-burst LTP is not blocked by propranolol, but LTP is blocked by the complete antagonist ICI 118551. Propanolol is an antagonist that blocks cAMP production but not ERK activation in response to isoproterenol [103], suggesting that the role of βAR activation in LTP is to promote ERK activation. In addition, genetic disruption of PKA anchoring to the β-adrenergic receptors produces deficits in both PKA phosphorylation of β-adrenergic receptors and ERK activation [102]. Our study uses a different approach to arrive at a similar conclusion that switching occurs in hippocampal neurons, a concept that may spur a novel line of research into alternative mechanisms of ERK activation underlying memory.

Our model suggests that ultrasensitive or switch-like behavior may be important for Gs-Gi switching in these neurons. Since the intracellular C-terminal tails of β_2_ARs have four consensus PKA phosphorylation sites [67], serines 261, 262, 345, and 346, it is plausible that the mechanism of switching is dependent on up to 4-site PKA phosphorylation of βARs, and thus we tested models of 1-, 2-, and 4-step phosphorylation of βARs. The sites were phosphorylated in a step-wise, or distributive, manner by PKA in the presence of phosphatase activity (see Table 5), conditions that are considered essential for producing switch-like behavior [64–66]. In addition, cooperativity between the phosphorylation sites was required, as the 1-site model did not exhibit switch-like behavior. These requirements are similar to those suggested for switch-like behavior at synapses [104].

The timing of receptor activation is important in determining the resultant cAMP signaling dynamics. When NMDA and isoproterenol are applied simultaneously, the induced cAMP response is synergistic (Fig 8A), similar to that reported in HEK293 cells [16]. However, as the experiments show, when isoproterenol precedes NMDA by several minutes, the cAMP response consistently is sublinear. Therefore, the signaling pathways activated by βAR depend on the temporal pattern of stimulation. βAR may enhance cAMP under some temporal conditions, and undergo switching and Gi production under other conditions. Thus, during β-LTP experiments, isoproterenol application may not be contributing cAMP and PKA activation. Instead, our results, together with those of [102], suggest that isoproterenol is enhancing ERK activation through Gi proteins. More importantly, βAR may be signaling through two different pathways in vivo. Thus, firing of noradrenergic locus coeruleus neurons just prior to CA3 neurons during behavior may enhance cAMP synergistically through Gs production, whereas the enhanced hippocampal memory formation by exogenous norepinephrine [72], or the chronic release of norepinephrine during stress [105] may be acting through the switching pathway.

## Supporting Information

S1 Data Excel spreadsheet of experimental data. The sheet labeled "Summary Tables" lists the response to NMDA and isoproterenol (as appropriate) for both soma and neurite. Each pharmacological condition is in a separate table. The sheet labeled "SAS format" lists the same data in a single table, appropriate for reading into the SAS software. The sheet labeled toltonegativi-div6-10 was used to generate the "Summary Tables". In addition to the information listed in "Summary Tables", it lists the response of individual neurites used to generate the average neurite response, and also lists the response of forskolin plus IBMX.
(XLS)

S1 File Compressed archive of model files (.xml files) and software (both simulation software written in java, and output-processing software written in python and C++) for running simulations. These files are identical to the files on modelDB.
(TGZ)
